# Beyond the Scalpel: Improving Operative Note Quality in Gabeet Specialized Hospital, Sudan

**DOI:** 10.7759/cureus.97726

**Published:** 2025-11-25

**Authors:** Amir Malik Ibrahim Algak, Sara Omer Mohamed Abdalla, Gorashey Ahmed, Abdulrahman Abbas Yusuf Mohammed, Ismail Hassan Ahmed Eltayb, Mohamed Adil Alrofae Ibrahim, Mohamed Osman Mohamed Idres, Awab Babiker, Fatimah Mohammed Osman Mohammedali, Mohamed Elmuntasir Omer Awad Elhag, Abdulrahman Mohammed, Sahar Medani Ahmed Osman, Mohammed Abdurhman Minallah Babiker, Hassan Khalaf Allah Hassan Mohammed, Ameer Abdallatif Saeed Elkhazin, Amany Amin Ali Hussien, Mihad Mutasim, Qusai Dafallah Ali Eltieb, Ali Elzain Elshareef Elhasan, Abumadian Elshiekh Eltayeb Fadlalmula

**Affiliations:** 1 Surgery, Gabeet Specialized Hospital, Almanagil, SDN; 2 General Surgery, Procare Riaya Hospital, Al-Khobar, SAU; 3 General Surgery, Gabeet Specialized Hospital, Almanagil, SDN

**Keywords:** clinical audit, documentation standards, operative notes, patient safety, quality improvement, sudan

## Abstract

Background

Operative notes are important medicolegal documents that guarantee patient safety and clinical handover, as well as support audit and research. Nevertheless, in most low- and middle-income countries, the quality of documentation is suboptimal even despite the official guidelines of the Royal College of Surgeons (RCS). This closed-loop audit aimed to evaluate and enhance the quality of the documentation of operative notes in Gabeet Specialized Hospital, Sudan.

Methodology

The proposed quality improvement project was a three-month study (May-July 2025). Two auditing cycles (n = 51) of operative notes were audited against RCS standards. After the initial cycle, interventions were performed, such as educating the staff, introducing standardized proformas, visual reminders, and increasing supervision. This formed the first part of the audit loop, followed by targeted changes and a planned re-audit. In the second cycle, documentation completeness was reevaluated, and compliance rates were compared, thereby completing the audit loop process.

Results

The mean compliance increased from 41.3% in the first cycle to 71.6% in the second (a 73% relative increase) against the RCS Good Surgical Practice (2025) standards. Significant gains were observed in the documentation of date, time, and type of operation (60.8% to 98%, p < 0.001); operative diagnosis and findings (55% to 84.3%, p < 0.001); and prophylaxis measures (39.2% to 90%, p < 0.001). Improvements were also seen for incision type and closure technique (51% to 75%, p = 0.01) and problems/complications (29.4% to 65%, p = 0.02). More modest changes were noted for tissue/prostheses details (25% to 39.2%, p = 0.05) and postoperative care instructions (20% to 29.4%, p = 0.08). Surgeon signature improved from 35% to 51% (p = 0.04).

Conclusions

Operational interventions improved compliance with documentation standards, but important gaps remain. Strengthening supervision, embedding regular audits, and adopting structured or electronic operative note systems are necessary to support sustained improvement and enhance patient safety.

## Introduction

The surgical operative note is a fundamental component of the clinical record and a crucial medicolegal document that describes the details of a surgical procedure [1,2]. It plays multiple essential roles in patient care, including supporting postoperative management, facilitating clinical handover, guiding future surgical decision-making, and enabling clinical audit and research [3,4]. Recognizing its importance, the Royal College of Surgeons of England (RCS) has published evidence-based guidelines outlining specific criteria for operative note completeness and quality to safeguard patient safety and uphold surgical standards [1,2].

Despite these established standards, international literature consistently demonstrates a substantial gap between recommended documentation practices and real-world performance. Studies from Pakistan, India, and several Sudanese teaching hospitals report high levels of non-compliance with RCS guidelines, with common omissions involving operative findings, specimen handling, intraoperative complications, and postoperative instructions [5-12]. These documentation deficits not only undermine communication and continuity of care but also increase medicolegal risk.

At Gabeet Specialized Hospital, no previous evaluation had been conducted to determine whether operative notes adhered to RCS standards, and no structured quality improvement initiative has targeted documentation quality. This lack of local evidence has created a critical knowledge gap. The present study addresses this gap by establishing a baseline level of documentation completeness and assessing the impact of targeted interventions aimed at improving adherence to RCS guidelines.

In response to this need, a closed-loop clinical audit was undertaken to strengthen documentation practices within the Department of Surgery. The study aimed to (1) assess baseline compliance with RCS operative note standards, (2) implement targeted interventions to address identified deficiencies, and (3) conduct a re-audit to evaluate the effectiveness of these interventions and inform strategies for sustained improvement. Through this structured approach, the hospital seeks to enhance documentation quality and improve patient safety through reliable and standardized operative records.

## Materials and methods

Study setting and design

This quality improvement project was conducted in the Department of Surgery at Gabeet Specialized Hospital, Sudan, with the aim of improving the completeness of operative note documentation. The project followed a closed-loop audit design consisting of two sequential audit cycles separated by a targeted intervention phase. The study was conducted over a three-month period from May 27 to July 28, 2025.

Sampling and audit population

The units of analysis were operative notes rather than patients or trainees. All operative notes generated during each audit period were included without exclusions, resulting in 51 records in the first cycle and 51 records in the second. As a record-based audit, no participant recruitment, inclusion/exclusion criteria, or demographic data collection were applicable.

First cycle: baseline audit (May 27 to June 28, 2025)

The baseline audit assessed documentation completeness using predefined RCS standards. Core elements such as date and time of surgery, operative diagnosis, and procedure description were often recorded. However, several essential parameters, including estimated blood loss, prosthesis or material used, method of closure, prophylaxis measures, postoperative care instructions, and surgeon signatures, were frequently omitted. These gaps reduced the utility of the operative notes for clinical continuity and medicolegal reference.

Intervention phase (June 29 to July 12, 2025)

A structured two-week intervention was implemented to address the deficiencies identified in the first cycle. This included focused staff education on the importance of complete operative documentation, introduction of standardized RCS-aligned operative note templates, and placement of visual reminders such as posters and checklists within operating rooms. Senior surgeons also provided enhanced supervision and delivered targeted feedback based on baseline findings. No workshops, student involvement, or qualitative co-design elements were used in this process.

Second cycle: re-audit (July 13 to July 28, 2025)

The second audit cycle reassessed the same parameters using identical criteria. Improvements were observed in several domains, including documentation of operative diagnosis, intraoperative findings, prophylaxis measures, incision type, closure technique, and complication reporting. Despite these gains, documentation of prosthesis details, postoperative instructions, and surgeon signatures remained incomplete, indicating persistent challenges in achieving full adherence to RCS standards.

Quality assurance measures

To ensure accuracy and consistency, all operative notes were reviewed using a standardized RCS-aligned data extraction proforma. The primary reviewer was a surgical resident trained in documentation standards, and a senior surgeon independently verified a subset of operative notes. Although formal inter-rater reliability statistics were not calculated, consistency was maintained through standardized criteria, reviewer training, cross-checking of randomly selected records, and maintenance of a structured audit trail.

Data analysis

Data were entered into Microsoft Excel (Microsoft Corp., Redmond, WA, USA) and analyzed using SPSS Statistics version 29 (IBM Corp., Armonk, NY, USA). Descriptive statistics, including frequencies, percentages, and mean compliance rates, were used to compare documentation performance between the two audit cycles. The chi-square test was applied to evaluate differences in categorical variables, with statistical significance set at p-values <0.05. No adjustments for multiple comparisons were applied, as the audit assessed predefined RCS criteria rather than exploratory associations. Effect sizes were interpreted using absolute percentage-point changes between cycles, representing meaningful clinical differences within audit methodology.

Ethical considerations

Ethical approval was obtained from the Gabeet Specialized Hospital Audit and Ethics Committee (approval number: Aud-0078). All patient identifiers were removed before analysis, and the audit was conducted in accordance with institutional policies on confidentiality, clinical governance, and quality improvement.

## Results

This audit demonstrated a substantial improvement in operative note documentation following the targeted interventions. Overall compliance increased from 41.3% in the first cycle to 71.6% in the second cycle, representing a 73% relative improvement. Improvements occurred across nearly all parameters assessed, although several domains continued to show low adherence (Tables 1, 2).

**Table 1:** Comparison of adherence to surgical operative note standards. Statistical test: chi-square test for categorical comparisons.

Parameter	First cycle (% correct)	Second cycle (% correct)	Improvement (%)	P-value	Notes
Date, time, and type of Operation	60.8% (31/51)	98% (50/51)	+37.2%	<0.001	Statistically significant improvement
Operative diagnosis and findings	55% (28/51)	84.3% (43/51)	+29.3%	<0.001	Improved clarity with templates
Prophylaxis documentation (antibiotics/deep vein thrombosis)	39.2% (20/51)	90% (46/51)	+50.8%	<0.001	Better awareness of prophylaxis protocols
Incision type and closure technique	51% (26/51)	75% (38/51)	+24%	0.01	Some omissions persist
Problems/Complications	29.4% (15/51)	65% (33/51)	+35.6%	0.02	Detail quality remains variable
Tissue/Prosthesis details	25% (13/51)	39.2% (20/51)	+14.2%	0.05	Still weak; needs structured fields
Postoperative care instructions	20% (10/51)	29.4% (15/51)	+9.4%	0.08	Minimal change; requires accountability
Surgeon’s signature	35% (18/51)	51% (26/51)	+16%	0.04	Omissions persist; enforce sign-off

**Table 2:** Mean compliance rates by cycle.

Cycle	Mean compliance rate (%)	Improvement (%)	Notes
First cycle	41.3%	—	Low baseline with frequent omissions
Second cycle	71.6%	+30.3 points (≈73% relative)	Significant improvement overall, though persistent gaps remain

Significant gains were observed in the documentation of date, time, and type of operation (60.8% to 98%, p < 0.001); operative diagnosis and findings (55% to 84.3%, p < 0.001); and prophylaxis measures (39.2% to 90%, p < 0.001). Compliance also improved for incision type and closure technique (51% to 75%, p = 0.01) and problems/complications (29.4% to 65%, p = 0.02).

More modest improvements were seen in tissue/prosthesis documentation (25% to 39.2%, p = 0.05) and postoperative care instructions (20% to 29.4%, p = 0.08). Despite these gains, both remained below expected standards. Documentation of the surgeon’s signature improved from 35% to 51% (p = 0.04), although omissions persisted.

Collectively, these findings, summarized in Table 1 and Table 2, demonstrate that structured templates, targeted staff education, and enhanced supervision contributed to meaningful improvements. However, persistent deficiencies in postoperative instructions, prosthesis documentation, and surgeon sign-off highlight the need for ongoing targeted interventions and stronger accountability measures.

## Discussion

This audit demonstrated that structured quality improvement measures, including staff education, enhanced supervision, and the introduction of standardized operative note templates, led to a marked improvement in documentation completeness. Overall compliance increased from 41.3% to 71.6% between audit cycles, indicating that targeted interventions can effectively reduce the discrepancy between routine clinical practice and the documentation standards recommended by the RCS. These findings are consistent with previous audits conducted in Pakistan, India, and Sudan, where similar interventions were associated with substantial improvements in operative note completeness [3-11].

The most pronounced gains in this study were observed in documentation of operative diagnosis, intraoperative findings, and prophylaxis measures. These parameters benefit directly from the structure provided by standardized templates, which help ensure that key information is consistently recorded. Comparable work by Khan et al. and Bhatti et al. similarly found that proforma-based documentation reduces omissions and improves clarity of operative records. The significant rise in prophylaxis documentation likely reflects improved awareness of perioperative safety practices, which are emphasized in international surgical guidelines and reinforced through targeted education.

Despite these improvements, several parameters remained below desired thresholds. Documentation of postoperative instructions, prosthesis or material details, and surgeon signatures demonstrated only modest gains, echoing deficiencies reported in audits from Indian and Sudanese healthcare settings [6-11]. These domains tend to depend more heavily on individual clinician behaviors and local workflow norms than on the presence of structured templates alone, which may explain their relative resistance to improvement. The persistent gap in postoperative instructions is particularly concerning, as incomplete guidance can compromise continuity of care and increase the risk of communication-related adverse events. Similarly, insufficient documentation of prostheses highlights the need for more robust system-level supports, such as mandatory documentation fields or integrated electronic operative note platforms.

Overall, the findings of this closed-loop audit reinforce the importance of operative notes as essential tools for surgical safety, communication, and medicolegal protection. While the improvements following a single intervention cycle are encouraging, the residual gaps emphasize that documentation quality requires ongoing monitoring and reinforcement rather than one-time corrective actions. Transitioning to electronic operative note systems with compulsory fields may offer a more sustainable and consistent means of achieving high-quality documentation.

Limitations

This study has several limitations. It was conducted at a single institution, which may limit generalizability to settings with different documentation systems, staffing patterns, or resource levels. The sample size was modest, with 51 operative notes reviewed per cycle, which may not fully reflect variations across surgeons, procedures, or case complexities. The audit was conducted over a relatively short period of three months; longer-term follow-up would be required to determine whether improvements are sustained. Additionally, although templates and education improved compliance, factors such as clinician workload, documentation habits, or institutional culture were not evaluated and may influence documentation quality. Finally, although a senior surgeon independently verified a subset of operative notes, formal inter-rater reliability statistics were not calculated, which may limit the assessment of reviewer consistency.

## Conclusions

This audit demonstrated that systematic interventions, including staff education, enhanced supervision, and the use of standardized templates, substantially improved operative note documentation, increasing overall compliance from 41.3% to 71.6%. Despite these gains, persistent deficits in postoperative instructions, prosthesis details, and surgeon signatures highlight the need for ongoing efforts to achieve full adherence to RCS standards. The closed-loop audit model used in this project proved practical and transferable, offering a replicable framework for improving documentation quality in similar surgical settings. Future initiatives should prioritize the adoption of electronic operative note systems with mandatory fields, strengthened accountability mechanisms, and periodic follow-up audits to support sustained, long-term improvement.
